# Beneficial Effects of Dark Chocolate for Episodic Memory in Healthy Young Adults: A Parallel-Groups Acute Intervention with a White Chocolate Control

**DOI:** 10.3390/nu12020483

**Published:** 2020-02-14

**Authors:** Daniel J. Lamport, Eleni Christodoulou, Christina Achilleos

**Affiliations:** School of Psychology and Clinical Language Sciences, University of Reading, Reading RG1 1AR, UK; eleni.christodoulou.19@ucl.ac.uk (E.C.); christina_a98@hotmail.com (C.A.)

**Keywords:** cocoa, flavonoids, polyphenols, cognition, cognitive function, memory, episodic memory

## Abstract

There is good evidence that cocoa flavonoids can acutely improve cognitive function in humans, possibly via mechanisms such as increased cerebral blood flow. To date, much of the evidence is based on measures of executive function with extracts and cocoa-based interventions with a high flavonoid content. The aim of the present study was to explore whether benefits to episodic verbal memory and mood are observed two hours post consumption of a commercially available dark chocolate (DC) bar relative to a 35 g white chocolate bar (WC). Ninety-eight healthy young adults (*n* = 57 females) aged 18–24 years consumed either a 35 g DC bar or a calorie-matched low flavonoid WC bar. Verbal episodic memory and mood were assessed pre consumption and 2 h post consumption. An ANOVA analysis showed that the DC was associated with better verbal memory performance for several outcome measures of the Rey Auditory Verbal Learning Test relative to the WC, however, there were no effects on mood. These findings lend support to the notion that everyday available portions of dark chocolate can confer benefits to the brain in healthy consumers.

## 1. Introduction

Dark chocolate contains a group of phytochemicals known as flavonoids, which naturally occur in many fruits and vegetables. The consumption of flavonoids and flavonoid-rich foods has been associated with numerous health benefits, and reviews of the literature indicate that cocoa flavonoids are associated with benefits to cardiovascular health [1] and cognitive function [2,3]. For example, chronic trials ranging from 8 weeks to three months show that daily consumption of flavonoid-rich cocoa is associated with positive effects on working memory and executive function in healthy older adults [4,5,6]. These chronic effects are accompanied by changes in cerebral blood flow [4,7] which is indicative of a cerebrovascular mechanism of action; for a review, see [8]. Changes in the brain-derived neurotrophic factor (BDNF), a protein associated with neuronal growth, have also been observed following twelve weeks of cocoa flavonoid consumption [9]. An acute cerebrovascular mechanism [10] also has potential to impact cognitive function in the immediate hours following cocoa flavonoid consumption. Indeed, a double-blind crossover design in healthy young adults [11] showed improvements in executive function up to 1 h following consumption of a flavonoid-rich cocoa drink relative to a low flavonoid control. Furthermore, improved spatial working memory in addition to benefits for visual contrast sensitivity two hours following dark chocolate consumption relative to a white chocolate control in healthy young adults has been shown [12], indicating that vascular benefits may extend to the retina. Moreover, dark chocolate bar consumption has also been associated with acute executive function improvements in adults aged 18–40 years [13] and these effects have been supported by changes in flow-mediated dilation [14] and changes in the concentration of oxygenated blood in the cortex, as measured with near-infrared spectroscopy in the immediate hours after consumption [15]. The majority of these supportive studies have administered pre-prepared chocolate drinks or dark chocolate bars containing concentrations of flavonoids (between 250–1000 mg), which are likely to be higher than that found in commercially available dark chocolate bars. Therefore, the focus of this research was to explore whether acute cognitive benefits are observed two hours post consumption of a 35 g dark chocolate bar commercially available in the UK, relative to a 35 g calorie-matched low flavonoid white chocolate bar. In support, benefits to non-cognitive outcomes, such as coronary circulation [16] and visual acuity/contrast sensitivity [17], have been seen following consumption of commercially available dark chocolate relative to a white or milk chocolate control. White chocolate is a viable comparator in that it has similar sensory properties to dark chocolate without the presence of flavonoids and can be matched for weight and calories. For consumers, it is also a realistic alternative to dark chocolate. The rationale for assessing performance two hours post consumption is based on evidence showing that this time frame reflects a period of absorption, digestion and metabolism of cocoa flavonoids for which vascular and behavioural effects are observed [10,11,12,13,14,15,16,17,18]. The aforementioned evidence indicates benefits predominantly for executive function and spatial memory, however, few studies have explored effects on verbal episodic memory. Cocoa-associated increases in hippocampal cerebral blood flow and concomitant benefits to spatial memory have been observed [4], and, given the important of the hippocampus for episodic memory, it is reasonable to hypothesise that cocoa-induced improvements may be seen for measures of verbal memory. Previously, subtle effects on subjective mood outcomes, such as fatigue [11,13] and contentment [18] following cocoa flavanols, have been seen; therefore, the present study also assesses subjective mood pre and post consumption. To summarize, the aim was to explore whether benefits to episodic verbal memory and mood are observed two hours post consumption of a commercially available dark chocolate bar relative to a white chocolate bar in healthy young adults.

## 2. Materials and Methods

### 2.1. Design

A parallel groups design was implemented with two treatments: dark chocolate (DC) and white chocolate (WC). Participants were assigned to treatments on arrival by the experimenter according to a predetermined alternating order generated by the principle investigator who did not collect any data (i.e., DC then WC then DC then WC). Outcome measures were assessed at two test sessions: pre consumption (Baseline) and two hours post consumption. Both treatments were matched for calories (203 kcal) and weight (35 g) and were commercially available 35 g organic products (produced by Green and Black’s ©, London, UK). The 35 g serving was chosen on the basis that this is a portion of chocolate typically consumed. The macronutrient content of the DC and WC treatments (respectively) was fat 15 g and 13 g; saturated fat 8.8 g and 8 g; carbohydrate 13 g and 18 g; sugars 10 g and 18 g; protein 3.2 g and 2.8 g; fibre 3.5 g and 0.01 g; salt 0.03 and 0.09 g. The DC was 70% cocoa content. The specific flavonoid content was not assessed. A database (http://phenol-explorer.eu/contents/food/439 accessed 15/08/2019) collating the analysis from multiple peer reviewed studies of dark chocolate using chromatography methods indicates a total flavonoid content of 237 mg/100 g which would equate to an 83 mg flavonoid dose for the DC treatment here. White chocolate contains no flavonoids [16]. This research was given a favourable opinion for conduct by the University of Reading School of Psychology and Clinical Language Sciences Research Ethics Committee (ref 2018-126-DL).

### 2.2. Participants

Ninety-eight healthy young adults (*n* = 57 females) were recruited (*n* = 49 DC and *n* = 49 WC) via opportunistic sampling from the University of Reading student population using posters and online advertisements. Exclusion criteria were any mental or physical illness, currently taking medication or supplements and smoking. Inclusion criteria were aged between 18–24, English speaker, and currently enrolled in a university degree programme. The latter enabled control over educational attainment, which is a predictor of episodic memory performance. Table 1 shows the demographic data broken down by treatment group. There were no significant differences between the treatment groups for any of the demographic variables (*p* > 0.05).

### 2.3. Outcome Measures

Rey’s Auditory Verbal Learning Task (AVLT) [19] is a well-established measure of episodic memory. Participants heard an auditory recording of 15 nouns (list A), read at 1 per second. Each presentation was followed by a free verbal recall of this list (recalls A1–A5). A new list of 15 nouns (list B) was introduced as an interference list on Trial 6 and was recalled once only (recall B). After a short delay (2 min), participants then free recalled List A without hearing the words. Two versions of the AVLT were used as previously described [20], which were counterbalanced across test sessions. A series of validated outcome measures were calculated according to Lezak [20] and previous research [21,22]: immediate word span (recall A1); words learnt (recall A5–A1); final acquisition (recall A5); total acquisition (sum A1 through A5); proactive interference (recall A1–B); retroactive interference (recall A5–A6); delayed recall (A6) and total recall (all trials).

Subjective mood was assessed with two questionnaires: the Positive and Negative Affect Schedule (PANAS) [23] and the Bond-Lader [24]. A computerised version of the PANAS was administered, which required a response using the keyboard based on the level to which participants were experience each adjective at that moment using a 5 point scale (1 very slightly or not at all; 2 a little; 3 moderately; 4 quite a bit; 5 very much). There are two outcome variables: (i) Positive Affect and (ii) Negative Affect, with a higher score representing higher levels of affect. The Bond-Lader is a series of 16 visual analogue scales (VAS) of 100 mm length, which requires the participant to indicate how they are feeling at that moment by marking the line. Each VAS is anchored by two adjectives (one at each end, e.g., alert–drowsy). There are three outcome variables: (i) Alertness, (ii) Contentment, and (iii) Anxiety, with higher scores indicating higher levels of each measure.

### 2.4. Procedures

All testing took place at the University of Reading School of Psychology in individual cubicles. Interested participants were checked for inclusion and exclusion criteria by email, phone call, or in person prior to attending the test day. Two hours prior to arrival on the test day, participants were required to fast from all food and drink except water. Informed consent was attained on arrival, at which point demographic measures were collected by a self-report questionnaire. Height and weight were measured by the experimenters. First, the Bond Lader was completed followed by PANAS and the AVLT. Participants then consumed the assigned treatment. Session 1 lasted approximately 30 min. Participants then left the laboratory and were required to remain fasted (expect water) until returning two hours later for Session 2. Session 2 followed the same procedure as Session 1, without the demographic measure assessment. Session 1 commenced anytime between 9 a.m. and 1 p.m. All data was collected between November 2018 and February 2019. Laboratory conditions, such as humidity, illuminance, and ambient temperature, were not assessed but remained constant throughout each test day. The experimenters were not blinded to the treatment on the test day, however, the mood and cognitive assessments were undertaken in a cubicle without the presence of the experimenter, and the responses were collected and subsequently coded by a computer to allow blinding for the analysis, which was undertaken by the principal investigator. All outcome variables were assessed with a 2 × 2 (Treatment*Session) mixed ANOVA. Significant interactions were clarified with post hoc independent *t*-tests comparing the two treatments at each time point, with a Bonferroni corrected alpha adjustment for type 1 error.

## 3. Results

As shown in Table 2, significant Treatment*Session interactions were observed for several episodic memory outcome measures, with the *t*-test showing that the DC was associated with benefits to episodic memory relative to WC at 2 h, whilst at the pre-consumption baseline there were no differences between the treatments.

**Figure 1:**
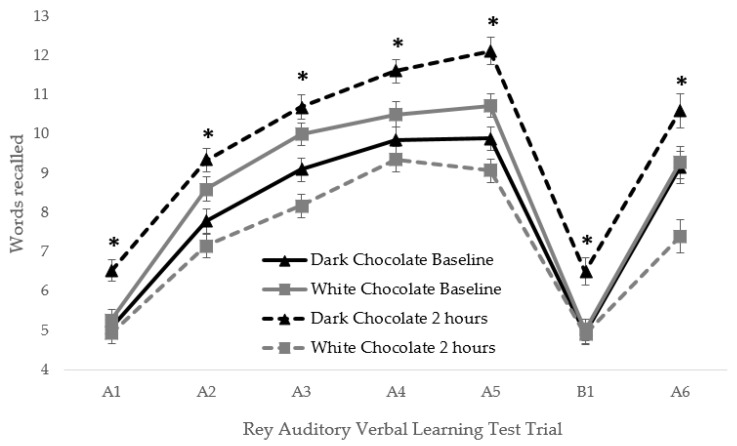
Words recalled (means and standard error) at each trial of the Rey Auditory Verbal Learning Task (AVLT) [19] at baseline and two hours post consumption for the dark chocolate (DC) (*n* = 49) and white chocolate (WC) (*n* = 49) treatment. An additional Treatment*Session*Trial ANOVA (2 × 2 × 7) revealed a significant three way interaction F (6576) = 4.06, p = 0.001. Post hoc *t*-tests at each trial were performed comparing DC and WC at baseline, and again at 2 h post consumption. A bonferroni correction for 14 *t*-tests was applied, and an alpha of <0.003 was considered statistically significant. Significantly better performance following DC relative to WC at 2 h post consumption was observed at all trials, as indicated by an asterix. No significant differences between treatments were seen at baseline.

Specifically, DC benefits were seen for immediate word span, words learnt, final acquisition, total acquisition, delayed recall, and total recall. Figure 1 also shows that the DC was beneficial for performance two hours post consumption relative to baseline, and relative to the WC control at each trial of the AVLT. No effects were observed for either measure of episodic memory interference, or any of the subjective mood measures. No effects of treatment were seen for any of the subjective mood outcomes. However, both positive affect and anxiety increased between baseline and 2 h post consumption whilst negative affect decreased between sessions (all *p* < 0.05; main effect of Session).

## 4. Discussion

The findings indicate that the consumption of a standard 35 g 70% cocoa dark chocolate bar can benefit verbal episodic memory relative to an equicaloric 35 g white chocolate bar two hours post consumption in healthy young adults. Previously, acute cognitive benefits in healthy young and middle-aged adults following cocoa consumption have been seen for measures of executive function and processing speed [11,12,13], spatial memory [12], and working memory [14]. This is one of the first reported observations of improved verbal episodic memory two hours post cocoa consumption. The benefit was notable in that seventeen more words were recalled following DC relative to WC over the course of seven trials. In support of the validity of the present findings, the task used (RAVLT) has shown sensitivity to other acute flavonoid interventions, including blueberries (see [25] for a review) and the Haskap berry [26].

The present data shows that recall was enhanced at all trials during the RAVLT following the DC relative to WC, indicating an increased capacity to learn words at initial presentation and throughout the task, including the short-delayed recall. Interestingly, there were no effects of interference, which suggests that the DC did not reduce the process of forgetting caused by the learning of new words or reduce the effect of old learning on new learning. The benefit of increasing one’s capacity to learn and recall words, with few effects on interference, is also seen in the blueberry flavonoid literature [25]. It would be of interest to explore effects on delayed recall ~30 min post learning and other complex subtleties of verbal episodic memory, such as source monitoring, which may give insight into the brain regions affected and the mechanisms of action. A possible mechanism of action within a two-hour timeframe is increased cerebral blood flow following flavonoid-rich cocoa, which has previously been demonstrated using MRI arterial spin labelling in resting state following a 493 mg flavanol cocoa drink in healthy older adults [10] and following a 519 mg flavanol drink in healthy young females [7]; for a review of cerebral blood flow effects following cocoa, see [8]. Of particular relevance for the memory benefits seen here are the observations of increased cerebral blood volume in a sub-region of the hippocampus (dentate gyrus) following 30 days of cocoa consumption, which indicates that areas of the brain associated with episodic memory are sensitive to cocoa flavanols. Indeed, flavonoids and their metabolites have been detected in the hippocampus and other areas associated with memory and learning, such as the cerebral cortex and cerebellum [27,28,29]. Increased cerebral blood flow following cocoa flavonoid ingestion may lead to acute cognitive benefits, such as improved episodic memory by a process of enhanced neurovascular coupling, whereby an increase in oxygen and glucose demand induced by increased neuronal activity during a cognitive task is met by increased blood flow to the region of activity due to the presence of cocoa flavonoids, via an mechanism of nitric oxide synthesis, which is a known pathway by which cocoa flavonoids can increase vasodilation [30]. In support of this, benefits to reaction time and concomitant increases in the fMRI BOLD (blood oxygen level–dependent) response in brain regions activated by an executive function task have been observed two hours post consumption of a 900 mg cocoa flavanol drink [15]. A notable outcome of the present research is that cognitive benefits were seen following an everyday portion of 70% cocoa dark chocolate. Whilst the exact flavonoid content is unknown, information from validated databases indicates it to be approximately 80–90 mg (see method), which is considerably lower than the majority of other acute cocoa flavonoid studies ranging from 250–1000 mg. This is encouraging for consumers and compliments epidemiological cross-sectional data showing positive associations between habitual chocolate intake and cognitive performance in 938 community-dwelling adults aged 23–98 [31] and in 2031 older adults aged 72 [32], both of which control for various cardiovascular, lifestyle, and dietary factors. Similarly, longitudinal data in 531 older adults (>65 years) reports a relationship between increased chocolate intake at initial assessment and a lower risk of cognitive decline several years later [33]. It would be of interest to extend these findings to clinical populations with memory impairments such as Mild Cognitive Impairment, in whom executive function benefits have been seen following eight weeks daily intake of a cocoa flavanol-rich drink [6]. Similarly, research into the chronic effects of everyday portions of DC on episodic memory in health adults would further our understanding of the association between evidence from clinical and epidemiological trials. The current study shows no DC benefits for subjective mood outcomes, which may indicate that the dose is insufficient, since other studies show reduced fatigue and higher contentment following >500 mg cocoa flavonoids (see [34] for an overview), however, it was interesting to observe an overall better mood state at Session Two relative to Session One, as indicated by an increase in positive affect and a decrease in negative affect. This may reflect the anticipation of test day completion and subsequently eating lunch given that participants had been fasted (notwithstanding the treatments) for at least four hours. Alternatively, this finding could reflect that simply consuming chocolate (DC or WC) was a positive experience and reduced negative feelings. The increase in anxiety between sessions was unexpected but could be due to negative anticipatory feelings associated with performing the RAVLT, as, upon completing the mood measures at Session One, participants were unfamiliar with the task. These explanations have no empirical support, and future research may benefit from assessing the sensory properties of the treatments. Indeed, a limitation is that the sensory properties may have contributed to the cognitive effects (e.g., taste/pleasure), however, this hypothesis is somewhat countered by an absence of differences in positive and negative affect between the DC and WC. A further limitation is the absence of an assessment of lifestyle factors, such as sleep quality, habitual diet, physical activity patterns, and hormonal variations, such as the menstrual cycle, which could have contributed to variance in cognitive function. Whilst a strength of this research is the commercial availability of the treatments, clearly, interpretations relating to the flavonoid content are limited in the absence of its assessment. Future research should, where possible attain the content of flavonoids and other active ingredients, such as theobromine and caffeine, (estimated here to be 390 mg and 35 mg, respectively, based on [35]). There is currently limited evidence for the individual contribution of theobromine for cognition [36], and reviews indicate caffeine doses >38 mg are require for discernible effects on cognition [37].

## 5. Conclusions

In summary, this research shows that 70% cocoa dark chocolate consumption can benefit verbal episodic memory two hours post consumption in healthy young adults relative to a white chocolate control. These findings support the notion that everyday available portions (35 g) of dark chocolate can confer benefits to the brain in healthy consumers.

## Figures and Tables

**Table 1:** Demographic information by treatment group.

	Dark Chocolate (*n* = 49)	White Chocolate (*n* = 49)	*p*-Value
Age (years)	20.7 (0.18)	20.6 (0.19)	0.59
Height (m)	1.71 (0.01)	1.71 (0.02)	0.99
Weight (kg)	64.6 (2.1)	64.9 (2.6)	0.95
BMI (kg/m^2^)	21.95 (0.57)	21.86 (0.59)	0.91
Sex (male: female)	22:27	19:30	0.54 ^

^ Pearson Chi Squared test.

**Table 2:** Means and standard error (words) for all outcomes with the Treatment*Session ANOVA interaction and post hoc *t*-test statistics comparing dark chocolate (*n* = 49) with white chocolate (*n* = 49) at each time point; baseline and two hours post consumption.

	Baseline Pre-Consumption			Two Hours Post-Consumption			Interaction *p*-Value
	Dark	White	*p*-Value *	Dark	White	*p*-Value *	
Immediate word span	5.08 (0.28)	5.29 (0.25)	0.59	6.53 (0.27)	4.94 (0.27)	<0.00001 *	0.00005 *
Words learnt	4.82 (0.38)	5.45 (0.27)	0.18	5.59 (0.32)	4.14 (0.35)	0.003	0.0004 *
Final Acquisition	9.9 (0.3)	10.7 (0.29)	0.051	12.1 (0.26)	9.1 (2.91)	<0.00001 *	<0.00001 *
Total Acquisition	41.7 (1.2)	45.1 (1.2)	0.048	50.3 (1.1)	38.7 (1.3)	<0.00001	<0.00001 *
Proactive Interference	0.14 (0.33)	0.27 (0.29)	0.779	0.02 (0.3)	0.02 (0.3)	0.999	0.852
Retroactive Interference	0.73 (0.32)	1.45 (0.35)	0.134	1.53 (0.31)	1.67 (0.37)	0.769	0.374
Delayed Recall	9.16 (0.39)	9.29 (0.44)	0.835	10.6 (0.41)	7.41 (0.47)	<0.00001 *	<0.00001 *
Total Recall	55.8 (1.6)	59.4 (1.7)	0.124	67.4 (1.5)	51.1 (1.9)	<0.00001 *	<0.00001 *
Positive Affect ^	33.71 (1)	33.78 (1)	0.96	34.86 (0.1)	35.43 (0.1)	0.69	0.633
Negative Affect ^	18 (0.65)	17.59 (0.7)	0.66	15.43 (0.7)	15.44 (0.7)	0.99	0.582
Alertness ~	68.8 (1.9)	62.9 (1.9)	0.03	73 (2.5)	67.2 (2.5)	0.11	0.99
Anxiety ~	34.2 (3.1)	30.3 (3.1)	0.38	41.4 (3.3)	37.9 (3.3)	0.44	0.95
Contentment ~	68.8 (2)	68 (2)	0.77	69.9 (2.1)	66.8 (2.1)	0.3	0.34

* With a Bonferroni correction for two *t*-tests, *p* < 0.025 is considered statistically significant. ^ As measured with the Positive and Negative Affect Schedule (scores range 10–50). ~ As measured with the Bond Lader Visual Analogue Scales (scores range 0–100).
